# Astigmatism research and application of vector method of the last half century: a bibliometric and visualized analysis

**DOI:** 10.3389/fmed.2025.1519487

**Published:** 2025-04-02

**Authors:** Jiamei Zhang, Lulu Xu, Mengyuan Shan, Liyun Yuan, Yi Dong, Yan Wang

**Affiliations:** ^1^Clinical College of Ophthalmology, Tianjin Medical University, Tianjin, China; ^2^Tianjin Key Laboratory of Ophthalmology and Visual Science, Tianjin Eye Institute, Tianjin Eye Hospital, Tianjin, China; ^3^School of Medicine, Nankai University, Tianjin, China; ^4^Nankai University Eye Institute, Nankai University, Tianjin, China

**Keywords:** astigmatism, vector method, surgery, bibliometric analysis, public health

## Abstract

**Background:**

As a common type of refractive error, astigmatism has always been one of the important factors affecting visual quality in clinical practice and it is quite an important public health burden. This study aims to evaluate astigmatism research and the clinical application of the vector method of the past half-century through a broad scientometric analysis, and to explore its reference value for guiding clinical treatment.

**Methods:**

The literature search was conducted on the Web of Science for astigmatic vector studies published from 1965 to August 2024. Retrieved publications were analyzed by the number of annual publications, prolific countries, and researchers, core publications and journals, and the number of citations through descriptive statistics. Collaboration networks and keyword analysis were visualized by VOSviewer and CiteSpace.

**Results:**

One thousand and fifty-nine publications were included for a visualized analysis. Vector methods help researchers to describe astigmatism specialized, and clinicians are mainly focused on the correction of astigmatism in both corneal and cataract surgery. Journal of Cataract and Refractive Surgery was the core journal for this field. The United States accounts for the largest proportion of publications and Australia had the highest citation ratio. Through the keyword analysis, the network identified 4 major research trends of corneal refractive surgery, penetrating keratoplasty, cataract surgery, and epidemiological surveys of astigmatism. “Photorefractive keratectomy,” “postoperative astigmatism” and “surgery” had significant burst strength and continuous attention to the astigmatism vector analysis.

**Conclusion:**

Vector analysis is the most commonly used method to evaluate astigmatism and could significantly improve the accuracy of astigmatism correction, particularly in areas of refractive and cataract surgery. The application of vector analysis is beneficial in guiding the design of surgical incisions, determining the nomogram, optimizing the surgical protocol, and improving the accuracy of astigmatism correction. Meanwhile, the popularization of vector method will help to improve the accuracy of astigmatism analysis and promote the benign development of public health.

## Introduction

Astigmatism is one of the common types of refractive errors in human eyes, which refers to the inconsistency of the refractive power of the cornea or lens in different meridian directions, resulting in defocus and blurred vision. Previous studies have reported that more than 80% of individuals with ametropia have different degrees of astigmatism, of which more than 25% have astigmatism greater than 1.00D (1, 2). Whether using lenses or surgery to correct astigmatism, or performing modern refractive cataract surgery, it is particularly important to assess the state of astigmatism. However, unlike myopia or hyperopia, the analysis and treatment of astigmatism is relatively difficult as it has both magnitude and axis. Arithmetic addition and subtraction were previously used or simply substituted with spherical equivalent (SE) for the residual cylinders before projected vector decomposition. However, the above methods cannot describe astigmatism in two dimensions. Astigmatism is usually divided into regular astigmatism and irregular astigmatism, while regular astigmatism can be further quantitatively analyzed by vector methods. Therefore, the vector method as an intuitive and accurate method, can provide more effective evaluation and help to achieve more perfect visual correction in clinical practice (3–6).

Postoperative astigmatism is one of the main causes of visual discomfort. It is the vector sum of the preoperative astigmatism and surgically induced astigmatism (SIA). The SIA concept was introduced by Jaffe and Clayman (7), as the astigmatic change induced on the corneal by a cataract incision whether it is small or large. It was calculated by employing cartesian coordinates and its axis was in the direction of the corneal steepening, as displayed on a polar diagram at half the orientation that is displayed on the double angle vector diagram. Then the innovation of Alpins (8) was to come up with a non-zero goal, and the newly introduced vector entities termed target induced astigmatism vector (TIA) and difference vector (DV). As one of pioneers in this area, several elements of Alpins’ approach have been incorporated into the recommendations of the Astigmatism Project Group of the American National Standards Institute (ANSI). Therefore, the Alpins method was adopted as the standard reference for the evaluation of safety and effectiveness astigmatism treatment by refractive lasers that reshape the cornea (9, 10). Later, Eydelman et al. changed Alpins terminology, raising the concern of confusion in the literature. For this, a subsequent editorial effectively examined the similarities and differences between the Eydelman and Alpins techniques, and set out in a detailed table, containing both the original Alpins Method terms alongside the ANSI altered terms together with the reasoning behind the preference of adhering to the original terminology. It was concluded that the source methodology and terminology of Alpins was recommended to be employed by the subsequent peer reviewed literature, to avoid confusion to retain consistency with the source (3, 8, 9, 11–13). Since some of these methodologies were inconsistent, and the trigonometry arithmetic of the vector analysis method itself is relatively difficult to understand, making it difficult for clinicians to evaluate the effectiveness of surgery (3, 10, 12).

Due to the complexity of the astigmatism analysis, it is necessary to integrate and summarize the related research. Bibliometric analysis can obtain sufficient and effective information, integrate research branches, and identify potential transformative papers so that clinicians can more intuitively understand the latest progress in a particular area (14–18). Given this, we summarized the general research on astigmatism using vector methods over the past half century, to analyze the hotspots, frontier progress, and core literature by using the bibliometrics method and citation network, to provide a comprehensive reference for clinical interests.

## Materials and methods

To ensure comprehensive and accurate data retrieval, this study utilized the Web of Science Core Collection (WoSCC) provided by Thomson Reuters (Philadelphia, PA, United States), limited to the Social Sciences Citation Index (SSCI) and Science Citation Index Expanded (SCI-EXPANDED). The retrieval strategy was as follows: TS = (astigmati*) AND TS = (vector*), covering the period from 1965 to 2024, with the cutoff on August 31, 2024. After removing duplicates, the retrieved papers were screened for eligibility. The publication type was restricted to articles, with no language limitations (Table 1).

**Table 1:** Summary of data source and selection.

Category	Specific standard requirements
Research database	Web of Science Core Collection
Citation indexes	SSCI and SCI-EXPANDED
Searching period	1965 to August 2024
Language	No restrict
Searching keywords	TS = (astigmati*) AND TS = (vector*)
Document type	Article
Data extraction	Export with “full record and cited references” in “plain text” format
Sample size	1,284

For knowledge mapping, VOSviewer (version 1.6.20, Leiden University, Leiden, Netherlands) and Citespace (version 6.3.R1, Drexel University, Philadelphia, PA, United States) were employed (19). VOSviewer constructs networks of scientific publications, journals, researchers, countries, keywords, or terms, using probability-based data standardization and offering various visualizations like network, overlay, and density views (20, 21). Items are linked via co-authorship, co-occurrence, or co-citation. The fractional counting method was used, ignoring publications with over 25 authors or countries (22).

Citespace, a Java-based software integrating scientometrics, visual analysis, and data mining, combines knowledge mapping with bibliometric analysis to identify research hotspots and trends, determine progress and frontiers, and predict field development (23). Together, these tools generate node-link maps for intuitive observation of research output and hotspots.

## Results

### Overall trends of publications

**Figure 1:**
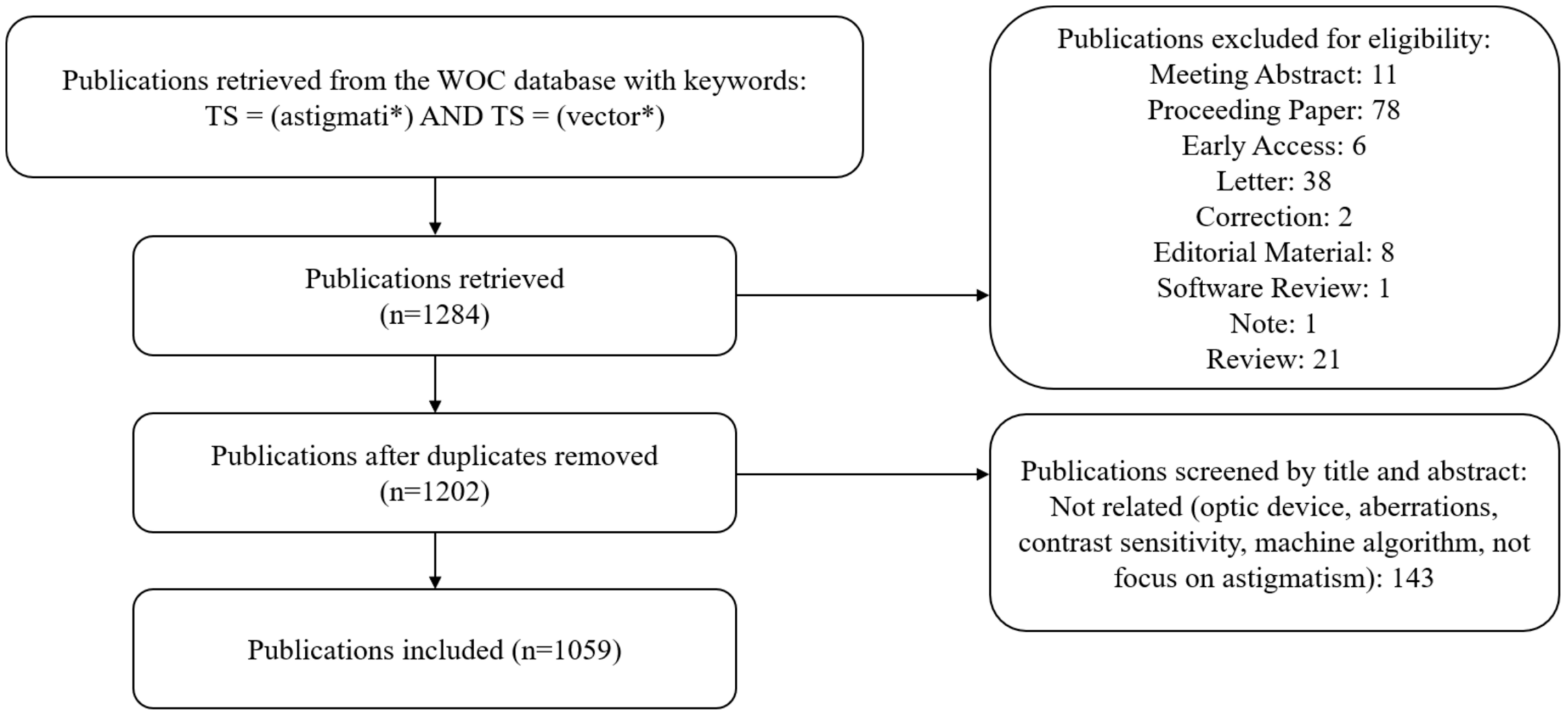
The flow chart of literature search.

**Figure 2:**
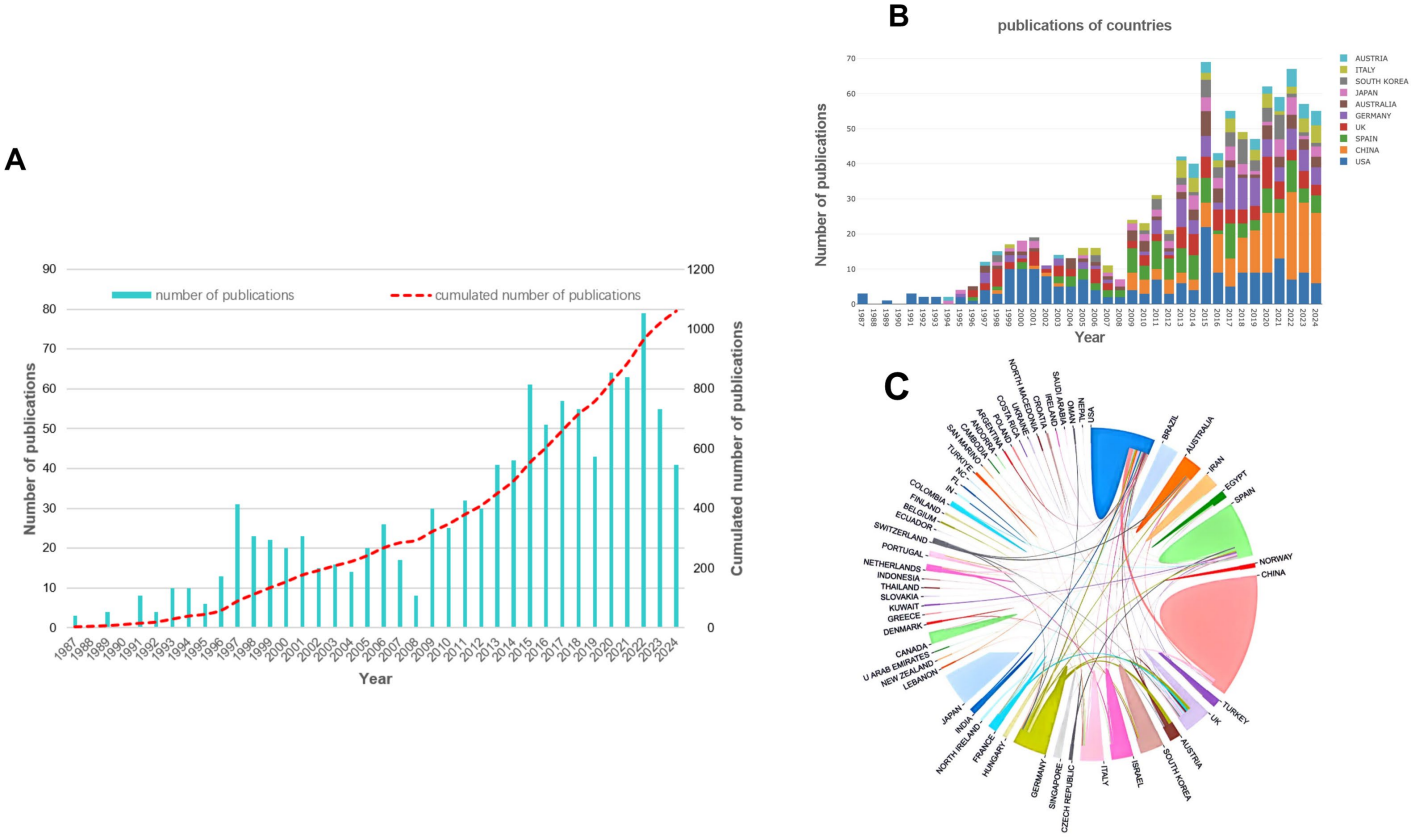
The annual number of publications from 1987 to 2024 **(A)**, the number of articles in the main countries over the years **(B)** and the chord chart of cooperative relationship between countries **(C)**.

After literature screening over the past half century, there were 1,059 publications finally retrieved from 89 journals, and 3,464 authors from 219 countries and regions were involved (Figure 1). Since 1987, the number of publications has increased gradually over the years. There were two peaks of publications, one of which occurred in the early 1990s, with the widespread popularity of laser *in situ* keratomileusis (LASIK), which raised concern about the effect of astigmatism correction. Another peak occurred after the widespread application of femtosecond laser in ophthalmology, which greatly improved the precision of surgery. The United States accounts for the largest proportion of publications, followed by China and Spain. The chord chart can more directly show the cooperative relationship between countries. The United States has collaborative relationships with many regions, the ties between China and the United States are the strongest, and European countries also maintain close cooperation (Figure 2). Citation ratio was defined as the average number of citations per article, Australia had the highest citation ratio, followed by the United States, Japan, Spain, and the UK, indicating that the articles from these five countries were more prone to hot topics and were discussed by more researchers (Table 2).

**Table 2:** The top 10 productive countries and their citation ratio in astigmatism vectorial research.

Rank	Country	Number of citations	Number of publications	Citation ratio
1	United States	5,773	212	27.2
2	China	2,124	170	12.5
3	Spain	2,792	111	25.2
4	United Kingdom	2,022	96	21.1
5	Germany	1,845	95	19.4
6	Australia	1,611	59	27.3
7	Japan	1,472	57	25.8
8	Italy	950	50	19.0
9	South Korea	867	50	17.3
10	Austria	516	38	13.6

### Distribution and co-citation network of publications, researchers, and journals

**Figure 3:**
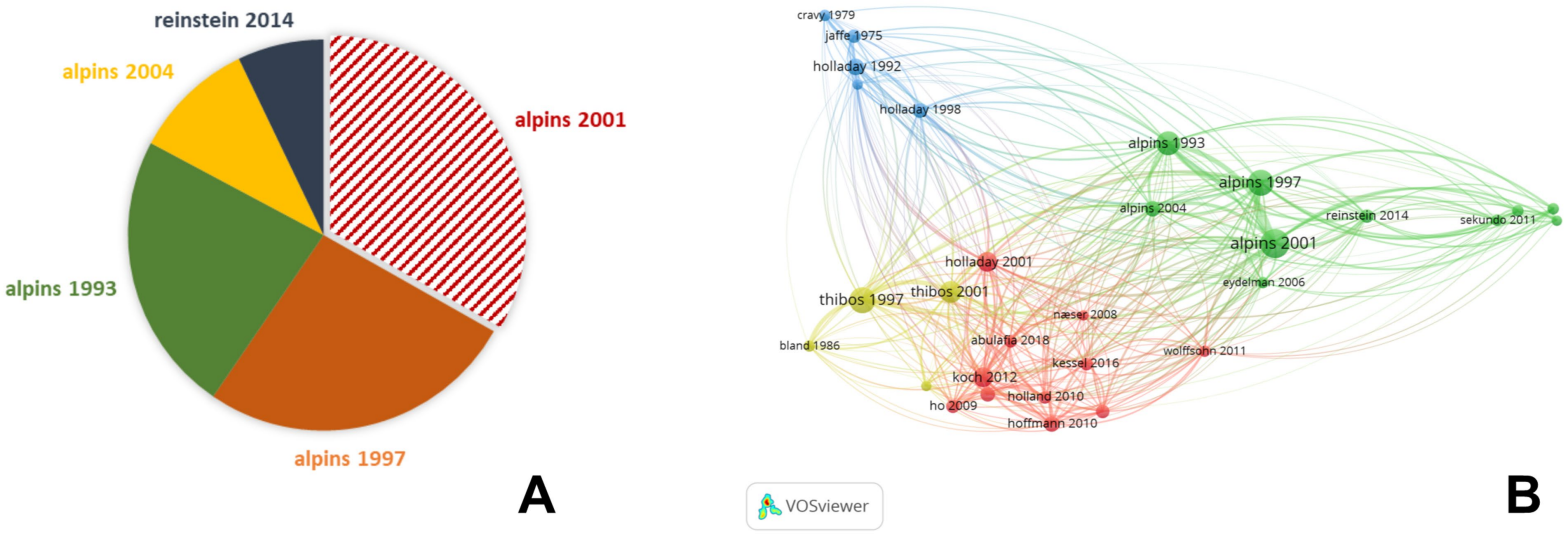
The top 5 publications with highest citation weight in the field of refractive surgery **(A)** and co-citation network map of publications in astigmatism vector analysis **(B)**.

The most frequently cited publications demonstrated the mainstream development of the field, reflecting their great importance in vector analysis of astigmatism. Among them, some publications were mainly focused on the decomposition method and several reported the results of astigmatism correction (24). Others focused on the distribution of astigmatism, the effect of toric intraocular lens (IOL) on astigmatism correction, and the influence of posterior corneal astigmatism on total astigmatism. Publications co-cited by multiple researchers were considered critical knowledge in the field of astigmatism research. In the network visualization, literature in the green cluster mainly focused on the research of astigmatism in the field of corneal refractive surgery, among which several papers published by Alpins accounted for a particularly significant weight, indicating that his research had a significant impact on this field. Other literature with high co-citation frequency appeared in the red cluster, which mainly concerned the effect of corneal astigmatism on the application of IOL in cataract surgery. In addition, though the number of literatures in the yellow cluster was small, the power vector method of representing and analyzing spherocylindrical refractive errors published by Thibos was still a widely recognized method that simplifies mathematical and statistical analysis of refractive errors by dealing with astigmatism magnitudes to two axes 45 degrees apart. In essence, the method proposed by Thibos focuses more on the decomposition and description of astigmatism, which is not a vector analysis of change as performed by Alpins Method. However, the method proposed by Alpins may provide detailed information on the assessment of changes in astigmatism. If any modification in astigmatism is required, then method proposed by Alpins may provide more valuable data (24). Alpins and Thibos analyses are not interchangeable when it comes to their mathematical results and interpretation (Figure 3).

**Figure 4:**
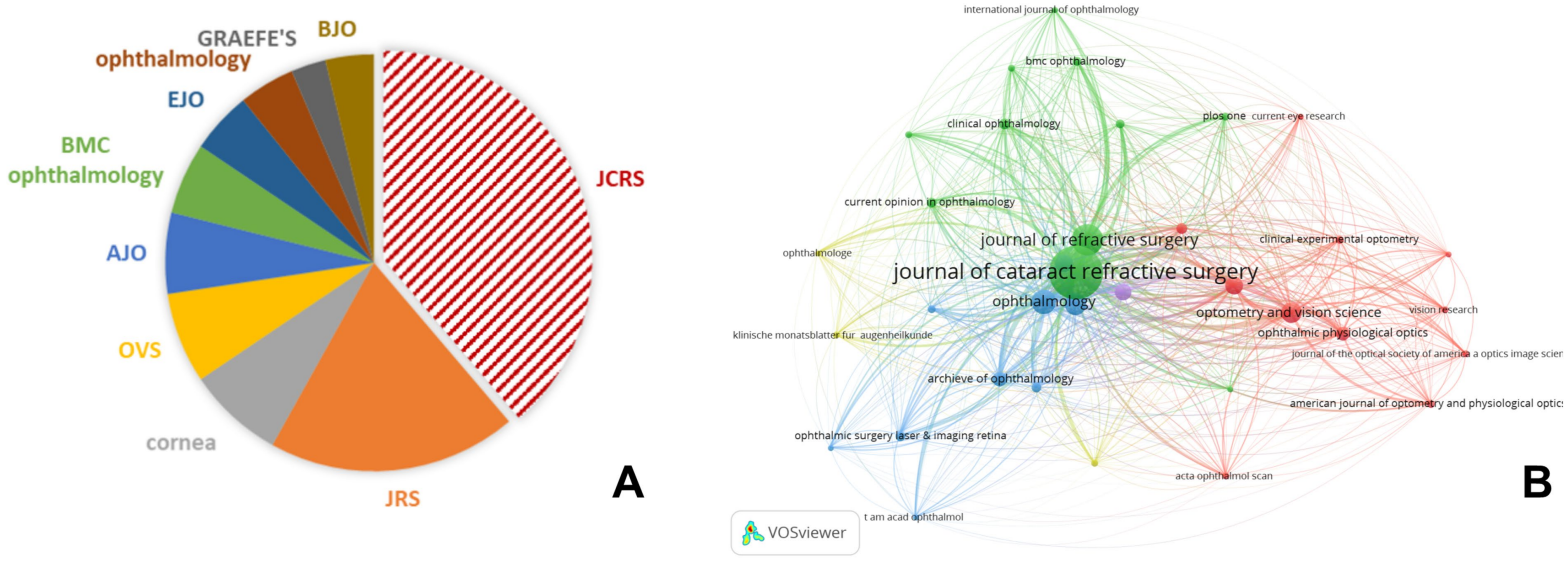
The top 10 journals with the most publications focused on astigmatism **(A)** and co-citation network map of journals in astigmatism vector analysis **(B)**. JCRS, journal of cataract and refractive surgery; JRS, journal of refractive surgery; OVS, optometry and vision science; AJO, American journal of ophthalmology; EJO, European journal of ophthalmology; GRAEFE’S, Graefes achieve for clinical experimental ophthalmology; BJO, British journal of ophthalmology.

In terms of the number of publications, the Journal of Cataract and Refractive Surgery (JCRS) published the largest number of related articles, followed by the Journal of Refractive Surgery and Cornea (Figure 4). The core journals of a subject defined by Bradford’s Law were identified by analyzing the number of articles published in the journals. According to Bradford’s Law, JCRS was the core source in the field of astigmatism analysis. These journals were mainly grouped into four main clusters in the visualized network map, with journals in the green cluster closely collaborating with specialized ophthalmology journals in the field of clinical applications and surgical techniques, especially corresponding to cataracts and refractive surgery. On the other hand, journals from the red cluster placed more emphasis on research related to optometry and optical research fields such as refractive error.

### Keywords of astigmatism vectorial research of astigmatism

**Figure 5:**
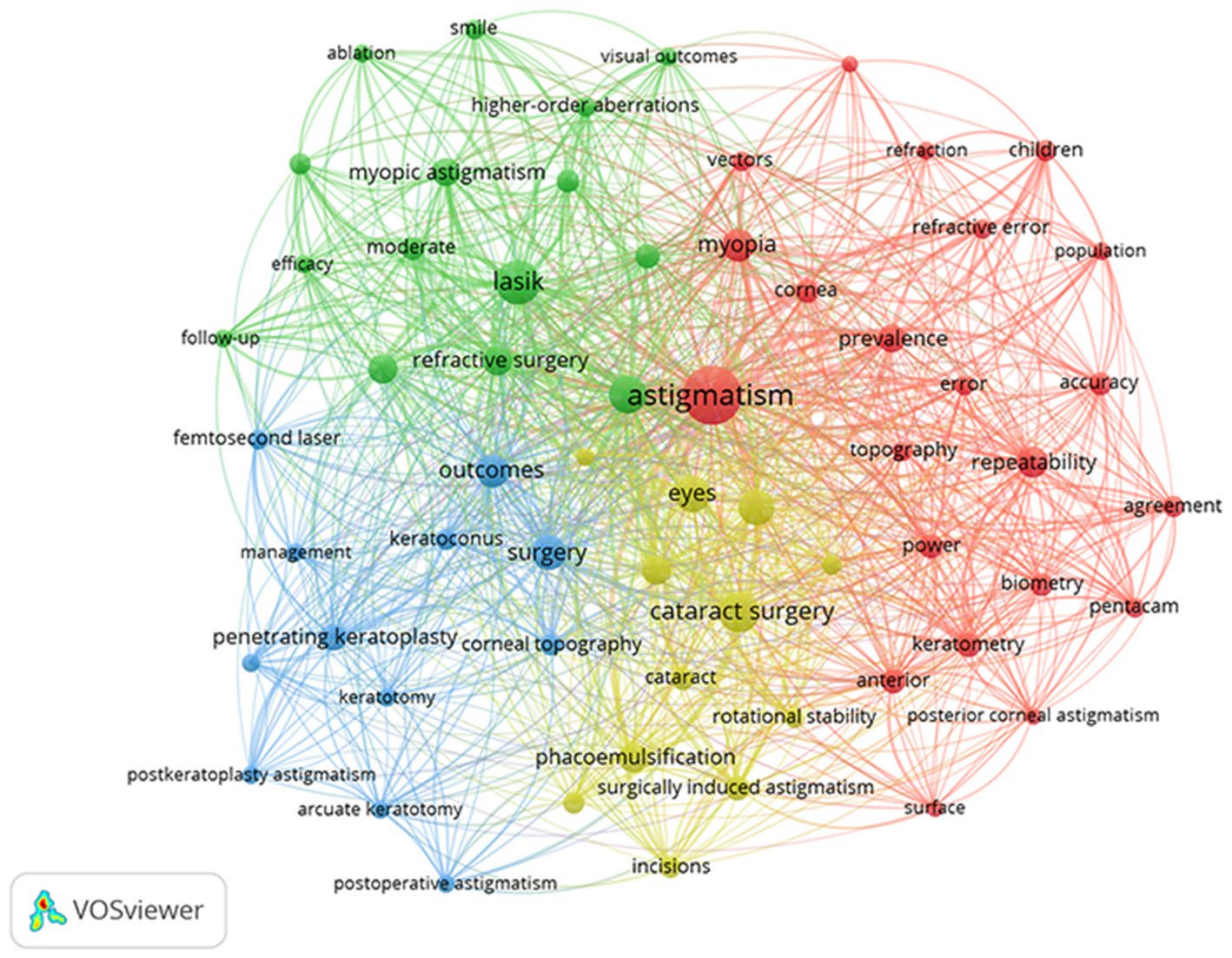
The co-occurrence network of keywords of astigmatism vectorial research.

Of the 2,082 keywords, synonyms were merged, and invalid words were excluded, then 58 keywords were visualized by network view which were grouped into 4 clusters (Figure 5). Keyword co-occurrence analysis can detect high-frequency keywords and the correlation between high-frequency keywords in a certain field during the statistical period, and reveal the current research hotspots. In the network visualization, the higher the weight of a keyword, the larger the point of the item. The closer two keywords are located to each other, the stronger their related by lines. The red cluster was the most prominent in the network view, in which “astigmatism,” “myopia,” “repeatability,” “prevalence,” and “power” were the core keywords of this cluster, indicating that this cluster mainly focuses on the astigmatism distribution in the ametropic population and the biological parameters and measurement for astigmatism. Another notable research hotspot focused on the analysis and effectiveness of astigmatism correction in corneal refractive surgery, in which “LASIK,” “vector analysis,” “refractive surgery,” “photorefractive keratectomy” and “myopic astigmatism” were the core keywords, represented by the green cluster. The analysis and management of astigmatism in cataract surgery were clustered in the yellow group, in which “cataract surgery,” “eyes,” “corneal astigmatism,” “SIA and flattening effect” were commonly used, and the analysis and management of penetrating keratoplasty were grouped in the blue cluster.

**Figure 6:**
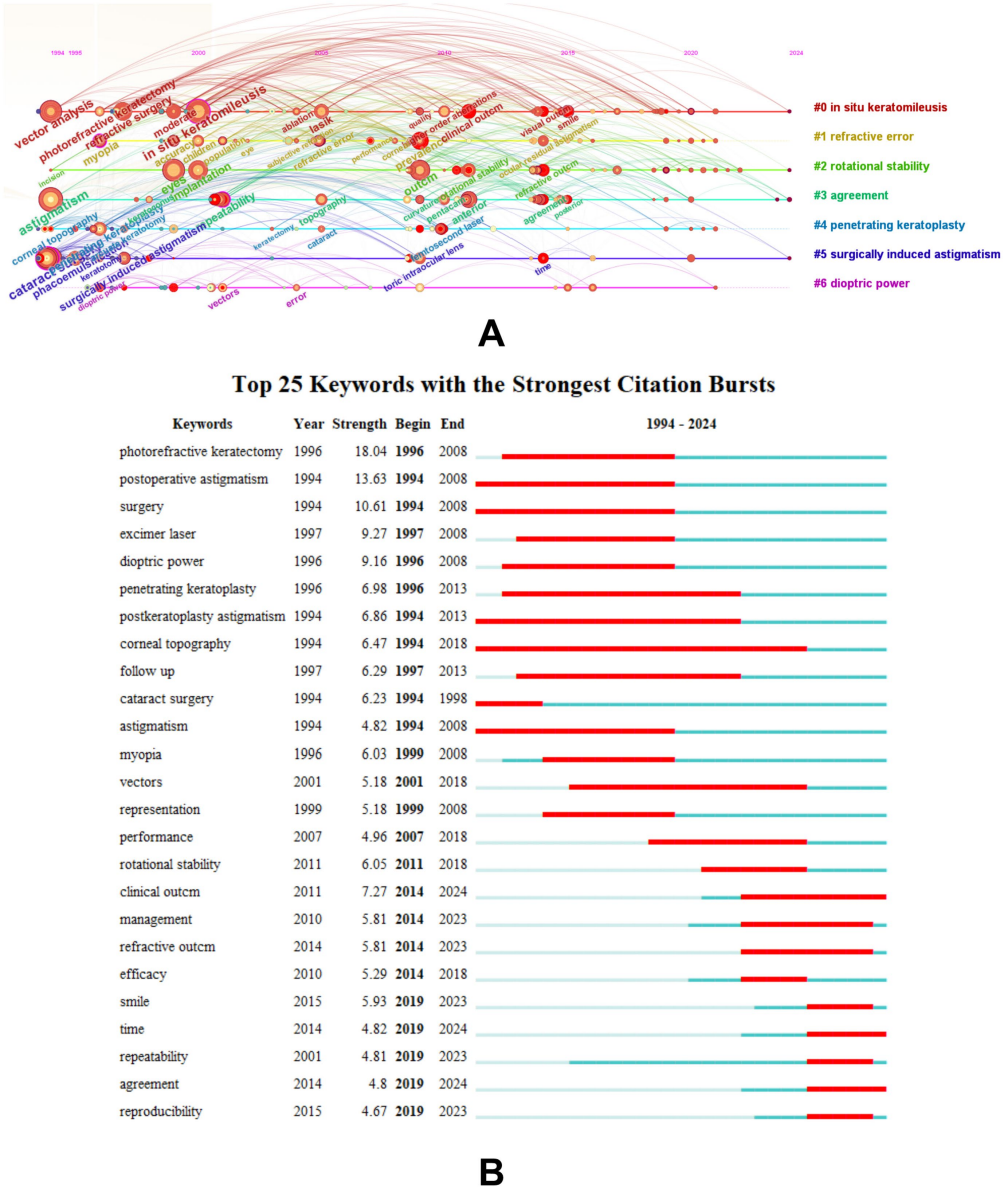
Timeline map of the keywords according to the first appearance year **(A)** and top 25 keywords with the strongest citation bursts on astigmatism research **(B)**.

The keyword cluster timeline map was based on cluster analysis, and the evolution of keywords under each cluster was demonstrated according to the first appearance year of the time slice. It can be seen that with the continuous improvement of corneal refractive surgery technology, the analysis of astigmatism has been focused on the three generations of PRK, LASIK, and SMILE. The quantitative description of astigmatism has gradually developed to focus on the repeatability and the agreement of astigmatism results. Through vector analysis, the understanding of astigmatism went from dioptric power gradually to the effect of ocular residual astigmatism (ORA) and posterior corneal astigmatism on vision. With the widespread use of toric IOL, reducing SIA to improve the accuracy of astigmatism correction has also become a hotspot (Figure 6A). A keyword burst analysis of the top 25 keywords with the strongest citation bursts reflected the research hotspot of a field changes over time (Figure 6B). Among these keywords, “photorefractive keratectomy” had the highest burst strength (18.04), followed by “postoperative astigmatism” and “surgery.” One of the most frequently used keywords in the last decade was “SMILE,” which is closely related to the gradual development of SMILE surgical techniques in recent years. During the latest development stage, most of the burst words focused on surgical methods and clinical management, indicating that the vector method was more important in evaluating the effect of astigmatism correction in clinical practice.

## Discussion

As a common type of refractive error, astigmatism has always been one of the important factors affecting visual quality in clinical practice. Before the application of vector methods, the study of astigmatism was simply a scalar description of the diopter. Vector analysis has been derived from the addition and subtraction of obliquely crossed cylinders known as Jackson cross-cylinders (JCC) theory since the 1990s, which states that any refraction result in the form of a spherical cylinder can be decomposed into a combination of an equivalent spherical lens and two cross-cylinders. There are a variety of calculation methods, including the sine and cosine law calculation method (25, 26), polar values method (27), dioptric power matrix (28), power vector analysis (5), and Alpins method of astigmatism analysis (3). Besides the astigmatism decomposition, the vector concept was flourished in the early 1980s. Jaffe and Clayman (7) proposed the SIA in 1975, which is regarded as the vector corrected by surgery, is the vector difference between the preoperative and the achieved astigmatism or cylinder at the corneal plane. From SIA, Alpins in 1993 introduced the non-zero target known as target astigmatism, which by its geometry added two additional vectors, the target induced astigmatism vector (TIA) and the difference vector (DV). With these three fundamental vectors, the refractive surgeon can clearly understand the surgical effect on astigmatism, which is particularly important for evaluating the effect of corneal refractive surgery and analyzing the induced astigmatism that comes from the incision of cataract surgery, by determining the Flattening and Torque Effect of the incision when the SIA is “off axis” from the TIA. In addition, the application of a double-angle vector diagram (DAVD) makes astigmatism in the axial range of 0–180° be expressed in the rectangular coordinate system of 0–360° so that the description of astigmatism and vectors are not limited to complicated numbers and is easily interpreted by anybody who has familiarity with high school trigonometry.

Due to the complexity of astigmatism itself and the various vector methods, traditional literature reviews are mainly literature descriptions and may be affected by the personal preferences of researchers, while bibliometric analysis can objectively cover a wide range of research and large-scale literature, and thus make it more suitable for revealing the research trends, hotspots, and development in the field of astigmatism. As a high-quality digital literature resource database, WOS has been accepted by many researchers and is considered to be the most suitable database for bibliometric analysis (16–18, 29). A bibliometric analysis of nearly a half-century of vector analysis shows that these methods were mainly used in corneal, refractive and cataract surgery, researchers paid more attention to the clinical reference of instructing and promoting astigmatism intervention and correction.

### The application of the vector method for astigmatism in corneal surgery

In the field of corneal refractive surgery, vector analysis was commonly used to evaluate the correction of astigmatism after surgery. Some two decades prior, Randleman et al. had made a list that provided a comprehensive assessment of the most cited articles and authors in refractive surgery and demonstrated key focuses in this field (30). In the field of refractive surgery, Alpins’s vector analysis method is clearly more meaningful than other astigmatism decomposition methods such as Thibos, because his method can provide more quantitative indicators of the change in the magnitude or direction of astigmatism. Keywords such as “LASIK,” “vector analysis,” and “photorefractive keratectomy” were highly weighted. The photorefractive keratectomy (PRK), which is the most original and classic corneal refractive surgery, had the highest burst intensity and is thus a hot topic in the research of astigmatism. Vector analysis of astigmatism outcomes after PRK and LASIK helps understand the surgical design and optimization (31–35). Recently, keratorefractive lenticule extraction (KLEx, e.g., SMILE, SILK and ATOS), are new technique for removing a refractive lenticule through a small incision, has become a more innovative method to correct myopia and myopic astigmatism. Since the first vector analysis of astigmatic correction after SMILE surgery was reported (36), some research has focused on the effectiveness of astigmatism and demonstrated a trend of under-correction, especially for high astigmatism (37–40). Several publications had compared the results between SMILE and FS-LASIK (41–44), as well as the effectiveness compared with wavefront-guided LASIK (45–49), the outcomes revealed a tendency toward under-correction in the SMILE groups for astigmatism correction. However when Vector Planning was employed in a study by Jun et al. (50) without any nomogram adjustment, the correction index (CI) which is SIA/TIA, was close to 1.0 when analyzed by both corneal and refractive parameters. An obvious advantage of the vector analysis method is that it can better explain whether the source of inadequate reduction of astigmatism is from the CI and magnitude of error (MofE), or the angle of error (AofE), which does not affect the SIA and also not the CI, and thus provide a reference for improving the surgical design. Given the vector results, controlling the rotation of the astigmatism axis and establishing a nomogram adjustment (TIA/SIA) based on the inverse of the CI, have instructive values for corneal refractive surgery to improve the accuracy of astigmatism correction.

“Penetrating keratoplasty,” “keratoconus” and “corneal topography” were the keywords with high frequency in another cluster of co-occurrence analysis, which was more focused on the management of astigmatism after corneal surgery. The term “penetrating keratoplasty” burst in the 1900s for vector research of astigmatism, surgical skill, incision suture, the alignment between the corneal graft and the implant bed, and the healing reaction of epithelium after the corneal keratoplasty will cause the change in refractive and keratometric parameters and lead to the occurrence of postoperative astigmatism. In some cases, severe astigmatism might influence the quality of life and thus the patient may require additional surgery to correct astigmatism (51). Effective management for post-keratoplasty astigmatism included arcuate keratotomy (AK) or limbal relaxing incisions, and topography-guided FS-LASIK (52–56). The standard vector analysis showed that the correction effect of corneal astigmatism was more preferable in this case from corneal parameters than that of refractive cylinder. In light of this phenomenon, adjusting the nomogram to improve the predictability of astigmatism after keratoplasty is necessary, by employing parallel both corneal and refractive analyses (3).

### The application of astigmatism vector analysis in cataract surgery

Another important area where vector methods were used to analyze astigmatism was cataract surgery. The visualized network revealed that “cataract surgery,” “eyes,” “corneal astigmatism,” “implantation” and “phacoemulsification” frequently occurred in large amounts of research. The exploration of astigmatism has always been the focus of clinical attention in the field of cataracts. As cataract surgery becomes more minimally invasive, the size and location of surgical incisions may cause a change in SIA and the Flattening Effect (FE) when the “off-axis” effect results in torque and less effective flattening by the SIA (57). Theoretically, making an incision along the steep meridian of the cornea can neutralize corneal astigmatism due to the FE (58). When the axis of the SIA is not orthogonal to the incision, then the SIA alone will overstate the effect at the incisional site which could result in systematic undercorrection of toric implant cylinder power (57). However, as the steep meridian is usually measured *in vivo*, the predictability of SIA can be slightly imprecise. In general, the temporal incision produced less SIA, while the superior incision produced larger SIA due to its proximity to the visual axis and possible influence of corneal neovascularization (59, 60). Recently, femtosecond laser-assisted phacoemulsification (FLACS) has achieved higher accuracy in making corneal incisions. However, studies have shown that there is no significant difference in SIA compared with traditional techniques, indicating that the SIA is individualized and related to many factors (61–64). Besides, applying a functional IOL requires more accuracy for the design of surgery, which is closely related to the condition of postoperative corneal astigmatism. According to the analysis of SIA by determining the trend of FE at any incision site, surgeons can choose more appropriate management to estimate incisional effect to achieve minimum postoperative astigmatism.

### The application of astigmatism vector analysis in optometry

The epidemiological characteristics of astigmatism, such as “myopic astigmatism,” “prevalence,” “repeatability” and “power,” can be described more accurately with the help of vector analysis. For the contact and spectacle lens industry, most are focus on the distribution, genesis and correction of astigmatism. When these studies describe astigmatism, most of them are based on astigmatism decomposition, which geometrically interpret spherocylindrical as power vectors. These methods are perfectly suited for descriptive and mathematical analysis of astigmatism. The resolved cylinder parameters commonly used in this field were J_0_ and J_45_ and many studies focus on the distribution of total refractive astigmatism, anterior and posterior corneal astigmatism, and ocular residual astigmatism (ORA) (59, 65–67). ORA is a useful corneal parameter to calculate, and choose the one with the smallest ORA as this corneal value is the best representation of refractive status of the eye since it is closest to the refractive cylinder (11, 68). According to astigmatism resolved power, the J_0_ power vector had a slightly positive value at birth but more negative values with increasing age. The J_45_ power vector values remained close to zero but variability increases at approximately 70 years of age. The results of vector analysis were mutually verified with clinical observations that the corneal horizontal meridian becomes steeper due to the weakening of eyelid pressure, so astigmatism in the elderly is often against-the-rule (69, 70). This suggests that astigmatism is a global visual health problem and its impact increases with age (71, 72). In addition, vector methods also play an important role in the application of contact lenses, a significant reduction was found in refractive J_0_ only in astigmatism, without changes in corneal J_0_ and J_45_, and orthokeratology did not induce refractive astigmatism in non-astigmatic subjects (73–76).

Although many clinical and theoretical studies have shown that the vector method could provide a more comprehensive description of change in astigmatism characteristics, the parameters being vectors are based on calculation. There is an important distinction made between the difference between astigmatism and vector. Although they share the same units of measurement which is diopters and degrees, they are quite dissimilar entities possessing different properties, that being astigmatisms and cylinders can only be measured while vectors can only be calculated. Even when astigmatisms are displayed as a mathematical construct on rectangular coordinates, which is a double angle diagram spanning 360 degrees from the 180 degrees on a polar diagram, they are still astigmatisms. Although vectors are mostly calculated on a double angle vector diagram (DAVD) and the summated vector mean (SVM) is also calculated in this way, it is not the real astigmatism (11, 77). In other words, the results do provide a valuable guide for clinical practice. Astigmatisms and cylinders are measurement results and vary according to the device used and the area of the cornea being measured. Calculation of the various ORA’s versus the common refractive cylinder which is the gold standard for the total astigmatism of an eye and its perception, provides the necessary lowest ORA as an indication of the corneal value best matched to refractive cylinder, so adjusting according to clinical practice (68). The theoretical effect can only be used as a reference for the surgeon to design the operation combining corneal and refractive parameters employing Vector Planning for this purpose to manage significant prevailing ORA. And evaluate the prognosis employing vector analysis to determine success and refine outcomes. It is worth noting that the actual correction effect also depends on external factors during the operation and the difference in postoperative healing between individuals.

In conclusion, vector analysis is the most commonly used method to evaluate astigmatism and could significantly improve the accuracy of astigmatism correction prior to embarking on surgery, particularly in areas of refractive and cataract surgery. Bibliometric analysis demonstrates that more and more attention is paid to the details of the description of astigmatism, and the accuracy of new surgical techniques and correction methods for astigmatism should be evaluated by vector means. The vector analysis provides a standardized way to assess and report astigmatism outcomes, on the other hand, the application of vector analysis is beneficial to guide the design of surgical incisions and ablations, determine the nomogram, and optimize the surgical protocol to address corneal and refractive parameters.

## Data Availability

The original contributions presented in the study are included in the article/supplementary material, further inquiries can be directed to the corresponding author.
